# The Emergence of Physiology and Form: Natural Selection Revisited

**DOI:** 10.3390/biology5020015

**Published:** 2016-04-01

**Authors:** John S. Torday

**Affiliations:** Evolutionary Medicine Program, University of California, Los Angeles, CA 90502, USA; jtorday@labiomed.org; Tel.: +1-310-222-8186; Fax: +1-310-222-3887

**Keywords:** Natural Selection, mechanotransduction, calcium, lipid, evolution, cell-cell interaction, fractal, ultimate, proximate

## Abstract

Natural Selection describes how species have evolved differentially, but it is descriptive, non-mechanistic. What mechanisms does Nature use to accomplish this feat? One known way in which ancient natural forces affect development, phylogeny and physiology is through gravitational effects that have evolved as mechanotransduction, seen in the lung, kidney and bone, linking as molecular homologies to skin and brain. Tracing the ontogenetic and phylogenetic changes that have facilitated mechanotransduction identifies specific homologous cell-types and functional molecular markers for lung homeostasis that reveal how and why complex physiologic traits have evolved from the unicellular to the multicellular state. Such data are reinforced by their reverse-evolutionary patterns in chronic degenerative diseases. The physiologic responses of model organisms like *Dictyostelium* and yeast to gravity provide deep comparative molecular phenotypic homologies, revealing mammalian Target of Rapamycin (mTOR) as the final common pathway for vertical integration of vertebrate physiologic evolution; mTOR integrates calcium/lipid epistatic balance as both the proximate and ultimate positive selection pressure for vertebrate physiologic evolution. The commonality of all vertebrate structure-function relationships can be reduced to calcium/lipid homeostatic regulation as the fractal unit of vertebrate physiology, demonstrating the primacy of the unicellular state as the fundament of physiologic evolution.

## 1. Introduction

When Darwin first enunciated his theory of evolution in On the Origin of Species [1], the mechanism he provided was Natural Selection. The inference was that there are “natural” forces at large that favor certain individuals within and between species over others, reflected by their reproductive success. That was a huge leap in the direction away from the Great Chain of Being, towards a more scientifically testable understanding of evolutionary change. In its time, Natural Selection was more closely aligned with Linnaeus’s efforts to categorize biological species using Binomial Nomenclature [2]. Natural Selection remains a metaphor for the specific underlying mechanisms that would explain the seeming systematic evolutionary changes in biologic form and function seen in ontogeny and phylogeny. The absence of specific mechanisms for Natural Selection still begs the question as to how and why physiologic traits emerged over the course of vertebrate evolution?

Needless to say, because of the dominance of Darwinian evolution theory over the course of the last century and a half, it is exceedingly difficult to find “exceptions that prove the rule” for an alternative to Natural Selection, let alone the language to express it. Yet I have been able to identify such a testable and refutable mechanism by seeing the arc of evolution from the unicellular to the multicellular state [3], encouraging me to present a different way of thinking about the evolution of vertebrate physiology.

The inference of the term Natural Selection is that somehow “nature” is enigmatically doing the choosing. But how does she actually do so? By reducing biologic mechanotransductive traits like that of bone, lung and kidney [4] to adaptations to gravity as the way in which the environment has fashioned physiology [4], the underlying mechanism can be realized [5]. By reducing gravitational effects on biological systems to their elements [4], applied to their ontogeny, phylogeny and pathophysiology [5], coevolved traits can then be identified at the cellular molecular level. In the aggregate, this systematic approach has helped to elucidate the how and why of evolution [6].

It seems reasonable to begin with ontogeny as the “author” of evolution since the integrated mechanism of embryologic development from zygote to newborn is well delineated at the cellular-molecular, tissue, organ and organismal levels [7,8]. And the consensus is that phylogeny is the end-result of the differential expression of developmental mechanisms [9]. Developmental biologists are all too familiar with the “plasticity” of the lung in utero, and it has previously been shown that this is largely due to fluid distension as a mechanistic homolog of gravity [10]. Since gravity is the oldest, ubiquitously consistent (magnitude, direction) natural force, it would seem prudent and reasonable to focus on that process as an archetype. In subsequent sections, I will show the functional homology between the lung, kidney and skin at the cellular-molecular level; a further reduction of physiology to model organisms (slime mold and yeast) based on microgravitational effects; the fractal nature of physiology and its origins in chemiosmosis, calcium/lipid homeostasis as the basis for self-referential self-organization.

## 2. Biologic Structures and Processes in an Evolutionary Context

### 2.1. The Cell as the Fundament for Natural Selection

The formation of the cell membrane delineates the internal and external environments. Endosymbiosis leads to the compartmentation of cellular functions. The key to understanding this mechanism lies in the transition from the blastula to the gastrula, since this is the stage of embryogenesis when the unilamellar cell membrane differentiates into the endoderm, mesoderm and ectoderm, the three germ layers that generate the entire embryo. It is during this phase that epigenetic information must be transmitted functionally to the germ layers. Lewis Wolpert, a leader in the field of developmental biology, has rightfully stated that “It is not birth, marriage, or death, but gastrulation, which is truly the most important time in your life” [11]. Cell-cell interactions determine embryonic patterns of morphogenesis, ultimately selecting for calcium/lipid epistatic balance, in part, as the driving force for novelty, the life cycle, senescence, and death as declining, and ultimately failed homeostasis [3]. Determining the origins of this mechanism, starting from the unicellular state is key to understanding the how and why of physiology. I have exploited the integrating effect of microgravity on both unicellular and multicellular organisms alike for this purpose, as follows.

### 2.2. Force of Gravity and Development

Physiologically, Parathyroid Hormone-related Protein (PTHrP) expression is central to mechanotransduction in a wide variety of tissues and organs, ranging from bone, to lung, kidney and uterus. PTHrP is primarily thought of as a calcium regulating paracrine hormone, and its calciotropic effects can be documented in each of these organs [12,13]. However, that is the overt, superficially evolved function of PTHrP; by delving into its roles in the development and homeostatic effects of this molecule, deep cellular-molecular interrelationships emerge [3]. In the lung and kidney its roles in angiogenesis and vasodilation become apparent by parsing out its structure-function effects [14,15]. However, it is in its determination of overall alveolar or glomerular phenotypic expression revealed when the PTHrP gene is deleted that its deeper role in vertebrate evolution can be appreciated [16,17]. The vertical integration of PTHrP in lung evolution is now well documented [5], including its direct causal regulation by gravity/mechanotransduction [4], largely seen in the existential vertebrate water-land transition [18]. Similarly, the role of PTHrP in glomerular evolution can be seen during this same era [5], integrating fluid and electrolyte balance by signaling to the mesangium. Equally important in this analytic approach is the way in which PTHrP interdigitates with other cellular-molecular physiologic properties, best seen in the alveolar wall [19]. PTHrP regulates lipid trafficking in lipofibroblasts (LIFs), binding to its cell surface receptor, stimulating Protein Kinase A [20]. The down-stream effect of this cell-cell interaction is increased expression of Adipocyte Differentiation Related Protein (ADRP), the molecule that facilitates the recruitment of neutral lipid from the alveolar microcirculation to the LIFs, and from the LIFs to the alveolar epithelial type II cell [21].

ADRP is a member of the PAT Family of proteins that regulate lipid homeostasis [22]. Lipid homeostatic control refers all the way back to the advent of cholesterol in the cell membranes of unicellular eukaryotes [3], facilitating locomotion, respiration and metabolism by rendering the plasmalemma thinner and more “plastic”, enabling increased gas exchange and endo-/exo-cytosis. Evolution of lipid homeostasis, beginning with the geochemistry of lipids in the primordial Earth environment [23,24,25], was critical for vertebrate evolution since it was exploited for regulating calcium homeostasis, which had become dysregulated when carbon dioxide rose in the atmosphere, forming carbonic acid in the oceans, leaching calcium from the bedrock [26]. In response, unicellular eukaryotes evolved peroxisomes, which utilize neutral lipids to buffer calcium leaking from the endoplasmic reticulum due to physiologic stress [27].

This “daisy chain” of interlinked mechanisms of physiologic development, regulation and homeostasis is not a tautologic, teleologic “just so story”. For example, Peroxisome Proliferator Activated Receptor gamma (PPARγ) is the determinant of adipocyte differentiation from muscle-related myofibroblasts. Cete, *et al.* [28] have shown that muscle stem cells will differentiate into adipocytes in 21% oxygen, but will remain myocytes in 6% oxygen, intimating the interrelationship between atmospheric oxygen and the evolutionary relationship between myofibroblasts and LIFs [29,30,31]. The functional significance of this property of LIFs came to light in studies of the role of the neutral lipids stored in LIFs [32]; the lipids act as a chemical “sink” for peroxides formed when LIFs are exposed to oxygen, intimating their functional origin in protecting the alveolar wall during the water-land transition. Such a self-protective feature is an exaptation that refers all the way back to aerosols in the primitive Earth atmosphere [26]. The development, phylogeny and pathophysiology of the lung are consistent with this scenario [33,34,35]. Moreover, when LIFs are cultured in isolation from alveolar type II cells they rapidly dedifferentiate into myofibroblasts, recapitulating their evolutionary origins, given that (a) myofibroblasts do not support alveolarization [33], and (b) the dedifferentiation of LIFs to myofibroblasts is characteristic of all chronic lung diseases, which are simplification processes, *i.e.*, the alveoli resort to their developmental and phylogenetic phenotype through reverse evolution.

The other cellular-molecular interconnection revealed by this empiric approach based on lung alveolar development and phylogeny is the role of prostanoids. Experimentally, when LIFs are cultured with neutral lipids, they readily absorb them, but do not release them unless they are co-cultured with alveolar type II (ATII) cells or their secreted products [36]. The ability to mobilize neutral lipids and transfer them to ATIIs for surfactant phospholipid production is the physiololgic fundament of alveolar physiology, recognized in descriptive biology as Ventilation-Perfusion Matching (V/Q matching) [37]. And the failure of lipid transit between LIFs and ATIIs in service to oxygenation causes the formation of lipid peroxides that are toxic (personal observation while performing such cell culture experiments). Experimental analysis of the ATII secretions revealed that the bioactivity resided in the lipid extract, which contained prostaglandins (PG) E2 and F2α [36]). However, when these two prostanoids were assayed for their capacities to cause secretion of neutral lipid by LIFs, only PGE2 was bioactive [36]. Moreover, the specific PGE2 receptor, EP2, was identified on LIFs [36], confirming that the mechanism of PGE2 action as a secretogogue for neutral lipids was receptor-mediated. The subsequent finding that stretching ATIIs increased PGE2 secretion [36] corroborated its physiologic role in the integrated regulation of alveolar surfactant phospholipid production. In retrospect, this property of PGE2, being secreted by LIFs should not have been surprising, given the lipid storage homology between the LIF and the adipocyte, since catecholamines regulate the secretion of fatty acids from the latter homeostatically [38,39,40], and beta adrenergic receptors critical for lung vascular regulation evolved from the mesoderm as LIFs [41].

And as a note added in proof for the predictive value of the cellular-molecular approach being exploited herein, the other component of the LIF that evolved to facilitate neutral lipid mobilization was the hormone leptin, which is necessary for the coordinate regulation of neutral lipid utilization by the ATII for surfactant synthesis [42]. Like the βAR and PGE2, leptin is also a product of the adipocyte that coordinates lipid metabolism body-wide [43]. Within the alveolar niche, it acts to facilitate metabolism by specifically aiding in the efficient on-demand production of lung surfactant phospholipids [44]. Not to mention the fact that all of these molecular traits are mechanosensitive, reflecting their atavistic homologies between surfactant and buoyancy in the swim bladder [5].

Reduced to its cellular-molecular elements, the basis for positive selection for PTHrP-PTHrP Receptor signaling, ADRP, catecholamine-βAdrenergic Receptor and leptin-leptin receptor signaling becomes readily apparent [5]. This is particularly true for endothermy [18], which has been hypothesized to have evolved as a vertical integration of these elements in response to intermittent hypoxia. Experimentally, disruption of any one of these components results in reversion of the LIF to its myofibroblastic ancestral phenotype and simplification of the alveoli back to their evolutionary origin in the faveoli of frogs [29,30,31], or the swim bladder of fish [45]. Conversely, in the case of the frog lung, treatment with leptin drove its differentiation towards that of the mammalian lung [46], demonstrating the predictive power of this model of ontogeny, phylogeny, and pathophysiology as evolution [47,48,49,50].

### 2.3. The Glomerulus: A Mechanotransductive Homolog of the Alveolus

Based on descriptive physiology, the lung and kidney bear no resemblance to one another. However, when looked at from the cellular-molecular developmental, phylogenetic, homeostatic, pathophysiologic perspective, PTHrP signaling can be seen as their structural-functional fundament. Like the ATII epithelial cells that line the alveolus, the glomerulus is lined by epithelial Podocytes that express PTHrP under stretch-regulatory control [51]. PTHrP secreted in response to fluid distension signals to the fibroblast-derived mesangium, which surrounds the glomerular capillaries, regulating fluid and electrolyte flow into the kidney tubules [52]. Physiologically, both the alveolus and glomerulus are under mechanotransductive control; developmentally, both the lung and kidney are responsible for the formation of amniotic fluid; moreover, failure of the kidney to produce urine results in decreased amniotic fluid, causing lung hypoplasia, attesting to the developmental interaction between the kidney and lung. Phylogenetically, evolution of both the lung and kidney were existential for adaptation to land. Shear stress to the microvessels of each caused the PTHrP Receptor gene duplication that amplified PTHrP signaling, resulting in the evolution of both organs [19]. Pathophysiologically, the lung-kidney interaction for the maintenance of allostasis is recognized as cardiopulmonary syndrome [53].

### 2.4. Skin as a Vertical Integration of Vertebrate Physiology

The skin is perhaps the most direct homolog of the unicellular plasmalemma, acting as the organ defining the internal and external milieus. In terms of evolved complex physiology, the skin is the most primitive organ of gas exchange, excreting fluids derived from the circulation as a filtrate, like the kidney. And it is now known that the brain evolved from the skin [54]. At the cellular-molecular level, there is a strong functional homology between the lung and skin, both forming molecular barriers against water loss and as anti-microbial barriers; such barrier functions are exaptations of geochemical properties of the primordial Earth [23,24,25]. The epithelium of the skin packages lipids and anti-microbial peptides to form lamellar bodies, which are secreted into the extracellular space, where they coalesce to form the stratum corneum, a physico-chemical barrier against water loss and microbial attack. The epithelial lining cells of the alveolus, the ATIIs, similarly package and secrete lamellar bodies composed of lipids and anti-microbial proteins into the extracellular space, where they form tubular myelin, an extracellular structural form of lung surfactant [55]. The physical conformation of the tubular myelin, and therefore its ability to reduce surface tension, is dependent upon the concentration of calcium in the alveolar hypophase [56,57]. The fact that PTHrP coordinates all of these functions and is calciotropic [58] infers that it is regulating the activity of the surfactant in the hypophase.

## 3. Model Organisms as Functional Homologs Revealed by the Molecular Physiologic Effects of Gravity

### 3.1. Yeast

An objective understanding of the significance of the homologies between the lung and kidney can ultimately be realized by examining the effects of mechanotransduction on model eukaryotic unicellular organisms. Yeast are among the most primitive eukaryotes. When Saccharomyces cerevisiae is exposed to microgravity it loses its ability to polarize and to bud [59]. The former is characterized by the inability to regulate calcium flux, whereas the latter is indicative of the inability to reproduce. Cell polarization reflects the control of calcium flux, the epitome of metabolic control; budding reflects the ability to reproduce, the raison d’etre for biologic existence [60]. Hence the foundational features of life are lost due to microgravity, indicating its essential role in the evolution of life as we know it. The coordinated effects of gravity on such basic physiologic principles of life are due to their effects on the cytoskeleton, the cellular framework [61], revealing that the cytoskeleton is more than just the way in which the organism structures itself. The cytoskeleton is the basis for all three cardinal states of being for the organism- homeostasis [62], meiosis [63] and mitosis [64].

### 3.2. Dictyostelium Discoideum

But how does the cytoskeleton determine the physiologic state of the organism? That realization derives from the study of the slime mold *Dictyostelium discoideum*, in which it has been discovered that the gene that determines whether the slime mold is in the amoeboid or colonial form is determined by the Target of Rapamycin or TOR gene [65]. The TOR gene regulates every property of the cell—ion flux, nutrient use, oxygen, physical force—by monitoring the cytoskeleton and acting accordingly [66].

## 4. Fractal Properties of Physiology

The reason why physiology exhibits holistic, unitary behavior is because it is fractal. That is to say, it is self-similar at every scale, due to the underlying, integrative mechanisms of cellular ontogeny, phylogeny and homeostasis [67]. This process is self-referential, all the way back in vertebrate phylogeny to its unicellular origins [68]. What are the integrating mechanisms that would account for the evolution of multicellular organisms from unicellular life? If they were known, they would provide fundamental insights to Natural Selection.

**Figure 1:**
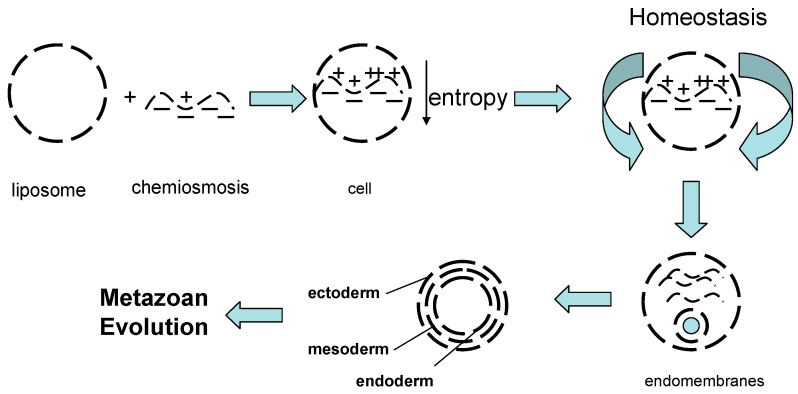
On the evolution of form and function. Starting with the formation of micelles and chemiosmosis, life began as the reduction in entropy, maintained by homeostasis. The generation of endomembranes (nuclear envelope, endoplasmic reticulum, golgi) created intracellular compartments; compartmentation gave rise to the germ lines (ectoderm, mesoderm endoderm) which monitor environmental changes and facilitate cellular Evolution accordingly. This is the basis for “fractal physiology”.

Elsewhere, it has been argued that the First Principles of Physiology are knowable since they are the mechanistic basis for ontogeny and phylogeny [9], which are one and the same process when seen from the cellular perspective, seemingly occurring on different time-scales, *i.e.*, they are diachronic [18,68]. Given that it is maintained that the innate organizing principle of physiology—homeostasis—is fractal [18,68]. We hypothesize that the epistatic balancing selection between calcium and lipid homeostasis was essential to the initial conditions for eukaryotic evolution, initiating the process of vertebrate evolution, continuously perpetuating and embellishing it from unicellular organisms to metazoans in all phyla (see Schematic, Figure 1) [5].

A classic misapprehension of the mechanism of evolution will be used to illustrate the difference between Darwinian mutation and selection, on the one hand, and cellular-molecular evolution from unicellular organisms on the other. In their essay “The Spandrels of San Marco and the Panglossian Paradigm” [69], Gould and Lewontin used the metaphor of the spandrels that were used in Byzantine architecture to fill in the gaps in the mosaic design in the central dome of St. Mark’s Cathedral, Venice, Italy, to disabuse their audience of the notion that everything in biology is purposeful, and that therefore there must be an underlying selection mechanism for them as well. However, if metazoans are derived from unicellular organisms, perhaps all metazoans are actually spandrels, derived from the organizing principles and mechanisms innate to unicellular life. For example, it is commonplace in the evolution literature for authors to point to evolved traits as “preadaptations”, the inference being that the trait pre-existed in an ancestral form. Carried to its logical extension, this would have led to the prediction that metazoans originated from unicellular organisms. In turn, if metazoans are derived from unicellular organisms, then by systematically determining the homologies that interconnect unicellular and multicellular organisms perhaps we can determine the underlying mechanisms involved.

### 4.1. Fractal Physiology—How and Why

Like Relativity Theory, the biology of multicellular organisms is also due to the interrelationships between space and time. In both cases, the Big Bang radiated out from its point of origin to give rise to both the physical and the biologic realms. In the case of the physical world, generating and then scattering the elements throughout the Universe, as evidenced by the so-called Redshift—when visible light or electromagnetic radiation from an object moves away from the observer, increasing in wavelength, shifting to the red end of the light spectrum. The background radiation of the Universe detected by radio telescopes exhibits such a Redshift, indicating that the Universe is expanding. The scattering of the elements based on their mass renders a structural hierarchy of “information”. By contrast, the biologic Big Bang was caused by the mimicking of that informational hierarchy of the physical Universe, biology generating an internal “pseudo-Universe” through the dynamic interactions generated by cellular information gathering, negentropy and homeostasis. Biologic “relativity” is due to the interactions between cells of different germline origins—ectoderm, endoderm, and mesoderm. The germline cells within the organism develop through interactions between one another, mediated by soluble growth factors signaling to their cognate receptors on the surfaces of neighboring cells. Those signaling motifs, or Gene Regulatory Networks (GRNs) are expressed as a function of their specific germlines, providing physical self-reference points for the physiologic internal environment to orient itself to the external physical environment. The genes take their “cues” from the interactions between the germline cells to generate form and function relative to the prevailing environmental conditions.

As a result, the organism can evolve in response to the ever-changing environmental conditions, the genes of the germline cells “remembering” previous iterations under which they (by definition) successfully mounted an adaptive response. Then, by recapitulating the germline-specific GRNs under newly-encountered conditions, they may form novel, phenotypically-adaptive structures and functions by recombining and permuting the old GRNs. This process is conventionally referred to as “emergent and contingent”. It explains how and why the same GRN can be exploited to generate different phenotypes as a function of the history of the organism, both as ontogeny (short-term history) and phylogeny (short-term history), the germlines orienting and adapting the internal environment to the external environment by expressing specific genetic traits.

For example, seen from the traditional perspective, this phenomenon is described as pleiotropy. However, once we realize that this is actually the consequence of an on-going process, and not merely a chance occurrence [70], the causal relationships become evident, offering the opportunity to understand how and why form and function have evolved. Such interactive, cellular-molecular mechanistic pathways project both forward and backward in time and space [3,5,18,34], offering the opportunity to understand our unicellular origins, and the functional homologs that form the basis for the First Principles of Physiology.

### 4.2. Fractal Physiology—In the Beginning

Life likely began with the formation of liposomes through the agitation of lipids in water (Figure 1) [71], bearing in mind that the moon separated from the Earth only 100 million years after the Earth was formed, offering billions of years for its effect on wave action to fashion life in this way. That interaction was chemiosmosis generating bioenergy, aiding and abetting negentropy, maintained by homeostasis. Endomembranes such as the nuclear envelope, endoplasmic reticulum, peroxisome and golgi apparatus formed intracellular compartments; compartmentation gave rise to the germ lines (ectoderm, mesoderm, endoderm) that monitor environmental changes and facilitate cellular evolution accordingly. These are the elements that fostered “fractal physiology” [9,67].

### 4.3. Fractal Properties of Physiology and Chemiosmosis

Peter Mitchell formulated his chemiosmotic hypothesis in 1961, essentially suggesting that the ATP synthesized in respiring cells comes from the electrochemical gradient across the inner membranes of mitochondria by using the energy of NADH and FADH2 formed from the breakdown of energy-rich molecules such as glucose [72].

Molecules such as glucose are metabolized to produce acetyl CoA as an energy-rich intermediate. The oxidation of acetyl CoA in the mitochondrial matrix is coupled to the reduction of carrier molecules such as NAD and FAD. The carriers pass electrons to the electron transport chain (ETC) of the inner mitochondrial membrane, passing them on to other proteins in the ETC. The energy available in the electrons is used to pump protons from the matrix across the inner mitochondrial membrane, storing energy as a transmembrane electrochemical gradient. The protons move back across the inner mitochondrial membrane through the enzyme ATP synthase. The flow of protons back into the matrix of the mitochondrion via ATP synthase provides enough energy for ADP to combine with inorganic phosphate to form ATP. The electrons and protons ultimately react with oxygen to form water.

### 4.4. Fractal Properties of Physiology and the Evolution of Calcium Homeostasis: From the First Cell to a Ubiquitous Intracellular Signaling System

With the advent of the first cell, the biological world split into extra- and intra-cellular spaces that immediately began controlling the ion content of the cell cytoplasm. The first cell was defined by a membrane composed of ion-conducting pores and ion pumps in combination with chemiosmosis—the movement of ions across a selectively-permeable membrane—as the source of energy to maintain entropy far from equilibrium. Failure of any of these components would have flooded the cytosol with calcium, testing the viability of the molecular machinery, potentially obviating the possibility of life [18,27].

The properties of the ion carriers were determined by the ionic composition of the primordial oceans. There were few dissolved ions present at the time—sodium, chloride, magnesium, calcium, potassium and other trace ions. The concentration of Ca^2+^ was the most critical to biology because it denatures proteins, lipids and nucleic acids alike. At non-physiological concentrations, Ca^2+^ causes aggregation of proteins and nucleic acids, damaging lipid membranes, and precipitating phosphates. High intracellular levels of Ca^2+^ are incompatible with life; at all phylogenetic stages, from bacteria to eukaryotes, excess cytosolic Ca^2+^ is cytotoxic. Consequently, the first forms of life required effective Ca^2+^ control, maintaining intracellular Ca^2+^ at effectively low concentrations—around 100 nanomolar, which by comparison is approximately 1–2 thousand orders of magnitude lower than that in the extracellular milieu. Indeed, even the most primitive bacteria are endowed with plasmalemmal Ca^2+^ pumps (which are structurally similar to eukaryotic P-type Ca^2+^ pumps of either the PMCA or the SERCA types) and Ca^2+^ /H^+^ and Na^+^/ Ca^2+^ exchangers [73].

Such high gradients of intra- *versus* extra-cellular Ca^2+^ characterize life ever since its inception. Maintenance of Ca^2+^ concentrations requires substantial energy consumption. Therefore it would have been surprising if evolution had not led to adaptation of Ca^2+^ homeostatic systems, whose initial functions were to protect the cell against massive, unrelenting Ca^2+^ pressure for ever-more complicated physiology.

## 5. Life has Authored Itself

**Figure 2:**
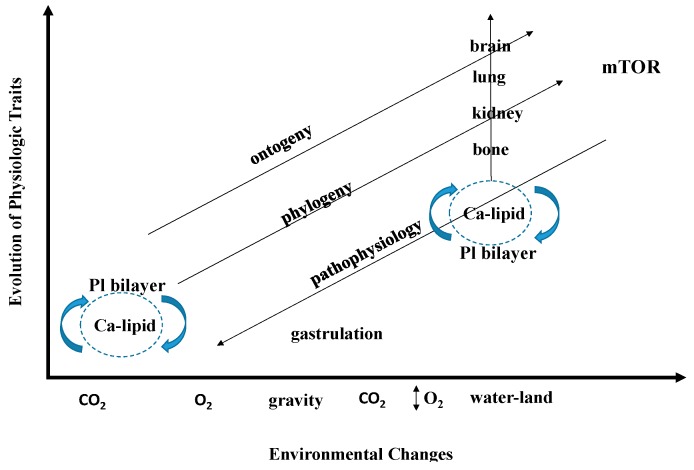
Physiologic evolution as one continuous Arc. from the inception of micelles from phospholipid bilayers (lower left corner, “Pl bilayer”) ontogeny, phylogeny and pathophysiology have formed parallel lines for the generation (or degeneration) of physiologic traits (Y-axis). Under selection pressure due to environmental changes (X-axis), exaptations have occurred, referring back to the unicellular First Principles of Physiology. This process of evolution gave rise to complex traits (bone, kidney, lung, brain), all of which funnel through the mammalian Target of Rapamycin (mTOR) gene, shown in the upper right.

The power of the cellular-molecular approach to the process and properties of evolution is that it allows a view from the origins of life to its physiologic complications as one continuous arc [3,5,18,35] (Figure 2). Moreover, that view provides insight to the exaptive changes [3,5,18,35] that have allowed for adaptation from earlier events in the organism’s history that have likewise been exapted, succeeding in sustaining and perpetuating it. Following that logic, one could feasibly trace life all the way back to its elemental origins. How would that look? Hypothetically, the polycyclic hydrocarbons in the primordial seas that originated in the asteroidal “snowballs” that formed them may have coalesced, and as the Sun warmed the Earth, such lipids would have liquefied, becoming more compliant. It has been postulated that lipids formed micelles, or liposomes [71], which are semi-permeable protocells, offering a protected space for chemiosmosis [72] and reduction in entropy, governed by homeostasis [67]. That scenario, when fast-forwarded, facilitated many of the existential changes in the evolution of eukaryotic vertebrates, including the nuclear envelope, endomembranes like the endoplasmic reticulum, the cholesterol-containing phospholipid bilayer, peroxisomes, barriers like the skin, the gas exchange organs initiated by cholesterol as surfactant [74], and even the brain [75]. This kind of phenomenology is usually referred to as self-organization or self-referencing [76], but is not usually vertically integrated in this way.

## 6. Conclusions

Novel, mechanistic ways of thinking about Natural Selection as a “Great Chain of Being”, starting from the bottom up have been provided. This approach is in marked distinction to Darwinian Descent with Modification, but of course Darwin had to contend with the prevailing paradigm in which royalty descended from G_d; ever since reading On the Origin of Species, I have assumed that it was exhaustive and lengthy because Darwin had to counterbalance the prevailing paradigm, the equally weighty and exhaustive King James Bible.

The current portrayal of the Great Chain of Being, starting from the spontaneously formed unicellular state moving forward to multicellular organisms, founded on first principles of physiology [18] offers the opportunity to integrate all aspects of biology into one continuous process, resulting from the interaction between the evolving organism and its environment [18]. With this self-organizing, self-referential set of principles in mind, scientific testing of causation rather than correlation and association is feasible.
